# Neural magnetic field dependent fMRI toward direct functional connectivity measurements: A phantom study

**DOI:** 10.1038/s41598-020-62277-4

**Published:** 2020-03-25

**Authors:** Yosuke Ito, Masahito Ueno, Tetsuo Kobayashi

**Affiliations:** Department of Electrical Engineering, Graduate School of Engineering, Kyoto University, Kyoto-Daigaku-Katsura, Nishikyo-ku, Kyoto, 615-8510 Japan

**Keywords:** Functional magnetic resonance imaging, Biomedical engineering

## Abstract

Recently, the main issue in neuroscience has been the imaging of the functional connectivity in the brain. No modality that can measure functional connectivity directly, however, has been developed yet. Here, we show the novel MRI sequence, called the partial spinlock sequence toward direct measurements of functional connectivity. This study investigates a probable measurement of phase differences directly associated with functional connectivity. By employing partial spinlock imaging, the neural magnetic field might influence the magnetic resonance signals. Using simulation and phantom studies to model the neural magnetic fields, we showed that magnetic resonance signals vary depending on the phase of an externally applied oscillating magnetic field with non-right flip angles. These results suggest that the partial spinlock sequence is a promising modality for functional connectivity measurements.

## Introduction

Comprehending brain activity is promising to conquer psychiatric and neurological disorders and develop emerging technologies, such as the brain-machine interface and neurocomputers. Many researchers, therefore, have been actively involved in unlocking the secret of brain activity, and developed many non-invasive techniques to measure brain activity. Non-invasive measurements of brain activity could be broadly divided into two categories. Electromagnetic methods detect the electric potentials and or magnetic fields generated by the electrical currents associated with local neuronal activity. The other group measures metabolic changes as a proxy for neuronal activities, such as oxygen and glucose concentrations. The former include electroencephalography (EEG)^1,2^ and magnetoencephalography (MEG)^3,4^, having fine temporal resolution but coarse spatial resolution. The latter include functional magnetic resonance imaging (fMRI)^5^ and functional near-infrared spectroscopy (fNIRS)^6^. In particular, fMRI based on blood oxygenation level-dependent (BOLD) signals is widely used for functional brain mapping due to its fine spatial resolution^5^. BOLD-fMRI, however, measures brain activity indirectly because it detects hemodynamic changes that follow the activation of neurons. Novel fMRI signals that measure neural magnetic fields directly, therefore, are desired.

Although several attempts were made to detect neural magnetic fields, none have demonstrated human neural magnetic field detection with MRI^7–15^. Such methods detect the phase shift or magnitude change of magnetisation correlating to the weak influence of neural magnetic fields on a static field. On the other hand, Witzel *et al*.^16^ and Halpern-Manners *et al*.^17^ reported neural magnetic field measurements with MRI instruments that exploit the spinlock sequence (Stimulus-Induced Rotary Saturation: SIRS). Based on the SIRS method, Jiang *et al*. proposed another spinlock sequence, called spinlock oscillatory excitation (SLOE)^18^. Truong *et al*. proposed yet another spinlock sequence for fMRI^19^. In this sequence, flip-back pulses are not applied in the SIRS sequence. These methods allow frequency selectivity by tuning the amplitude of spinlock pulses. The methods, therefore, could selectively detect rhythmic oscillations of neural magnetic fields such as alpha waves (8–13 Hz) and gamma waves (25–150 Hz)^20^. Furthermore, functional connectivity is a focus of interest in neuroscience, as diffusion MRI made it possible to analyse and clarify the anatomical association of white matter^21,22^. The resting-state fMRI (rs fMRI) was a popular technique to investigate the default mode network (DMN)^23,24^. The DMN was thought to be related to some neurological and psychiatric disorders^25–27^. Consequently, interest grew in the fundamental function along with the connectivity among different regions in the brain. Now, we focus on the functional connectivity in some tasks. In such a case, the time course of the processing in the brain should be observed. The method to observe the functional connectivity, therefore, should detect the frequency and phase of the signals. The SLOE can detect the initial phase of the oscillating magnetic fields^28^. The results, however, showed that the initial phase was 0° or 180°. In the previous study, we measured oscillating fields from a phantom via the spinlock sequence, SIRS, and studied the potential applications of this method to fMRI^29,30^. The weakest magnetic signals we could detect by the spinlock sequence were about 200 pT, which was in the range of the reasonably assessed value of the neural magnetic fields^29^. The SIRS, however, is insensitive to the signal phase.

This study proposes to modify the SIRS with non-right *α* and −*α* RF pulses, called the partial spinlock sequence, and clarify the effect of the phase of the oscillating fields on the MR signals acquired by the partial spinlock sequence. This method can measure phase differences among multiple sources by projecting the phase information to the MR signal intensities. We, therefore, will acquire the coherency of the neural magnetic fields, which leads to unravelling the functional connectivity, combining the frequency selectivity of the SIRS. We, therefore, simulated the magnetisation behaviour with the Bloch equation, including phase effects and performed phantom measurements with a loop-coil phantom and current-dipole phantoms.

## Methods

### Sequence and behaviour of magnetisation

**Figure 1:**
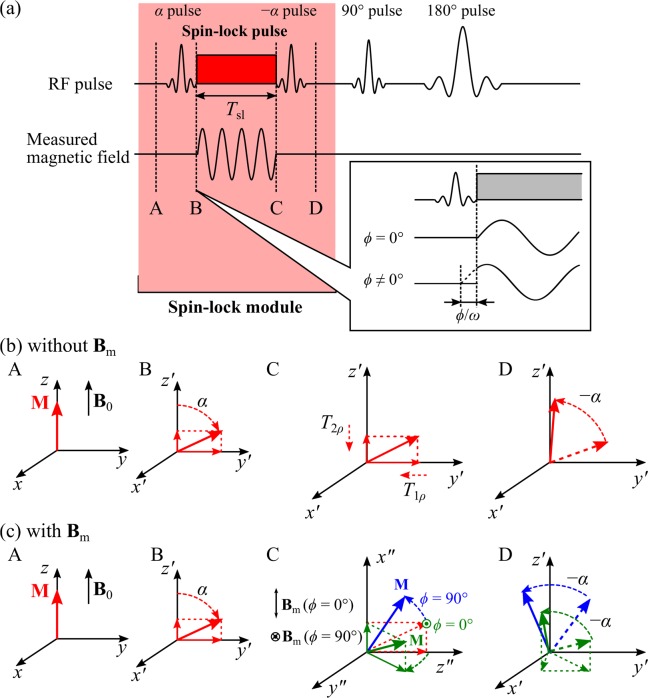
Partial spinlock sequence (**a**) and magnetisation behaviour without measured magnetic fields (**b**) and with measured magnetic fields of the phase difference between the measured magnetic field and the spinlock pulse *ϕ* = 0° and 90° as examples (**c**).

 Figure 1(a) shows the partial spinlock sequence based on the conventional spin-echo sequence^29^. This partial spinlock sequence consists of a spinlock module followed by a spin-echo sequence. The spinlock module includes an *α* RF pulse, a spinlock pulse, and a −*α* RF pulse, *α* is the flip angle. In the reference^30^, we provided the gradient pulses in the sequence diagram explicitly. We, however, leave out the display of the gradient pulses in this article since those do not significantly affect the behaviour of the magnetisation. When a static field **B**_0_ is applied in the *z*-direction, as shown in Fig. 1(b), the ± *α*RF pulses are applied in the *x*-direction and the spinlock pulse is applied in the *y*-direction. *T*_sl_ reveals the duration of the spinlock pulse. Figure 1(b,c) illustrate the behaviour of the magnetisation **M** when the flip angle is *α* without measured magnetic fields and with measured magnetic fields, respectively. $(x{\prime} y{\prime} z{\prime} )$ represents the rotating frame.

The magnetisation of protons in the static fields **B**_0_ is oriented in the *z*-direction (Fig. 1(b)A). Then, the magnetisation is rotated about the $x{\prime}$-direction by an RF pulse with the angular frequency ${\omega }_{0}=\gamma \left|{{\bf{B}}}_{0}\right|$. *γ* is the gyromagnetic ratio of protons. When an RF pulse is applied, the magnetisation is tilted from the $z{\prime}$-axis by *α*. The magnetisation, therefore, includes $y{\prime}$ and $z{\prime}$ components (Fig. 1(b)B). After that, the spinlock pulse applied in the *y*-direction holds the $y{\prime}$ component of the magnetisation in the $y{\prime}$-axis. We, therefore refer to the sequence as a partial spinlock sequence. Within the duration of the applied spinlock pulse, the $z{\prime}$ and $y{\prime}$ components of the magnetisation undergo *T*_1*ρ*_ and *T*_2*ρ*_ relaxation, respectively (Fig. 1(b)C)^30^. Finally, the −*α* pulse flips the magnetisation up toward the *z*-direction (Fig. 1(b)D). Meanwhile, when the measured magnetic field with angular frequency *ω* = *ω*_sl_ = *γB*_sl_ is applied, a secondary magnetic resonance arises between the measured magnetic field and the magnetisation while the spinlock pulse is applied. Here, *B*_sl_ is the amplitude of the spinlock pulse. In other words, in the rotating frame, the spinlock pulse acts as a pseudo-static field and the measured magnetic field acts as an RF pulse. The magnetisation, therefore, flips into the $z{\prime} x{\prime}$ plane. The flip angle is determined by the amplitude of the measured magnetic field, *B*_m_. The oscillating magnetic field **B**_m_ is defined as:

1 $${{\bf{B}}}_{{\rm{m}}}={B}_{{\rm{m}}}\sin (\omega t+\phi ){{\bf{e}}}_{z}.$$

Here, we consider the behaviour of the magnetisation in the doubly rotating frame with the rotating frequency *ω*_sl_/2*π*, replacing $x{\prime}$ by *y″*, $y{\prime}$ by *z″*, and $z{\prime}$ by *x″*. In the doubly rotating frame, the oscillating magnetic fields can be replaced by:

2 $${{{\bf{B}}}^{{\prime\prime} }}_{{\rm{m}}}={B}_{{\rm{m}}}\sin \ (\omega t+\phi )(\sin \,{\omega }_{{\rm{sl}}}t{{{\bf{e}}}^{{\prime\prime} }}_{x}+\cos \,{\omega }_{{\rm{sl}}}t{{{\bf{e}}}^{{\prime\prime} }}_{y}).$$

Consider the case *ω* = *ω*_sl_ introducing the rotating wave approximation, we obtain:

3 $${{{\bf{B}}}^{{\prime\prime} }}_{{\rm{m}}}=\frac{{B}_{{\rm{m}}}}{2}\left(\cos \ \phi {{{\bf{e}}}^{{\prime\prime} }}_{x}+\sin \ \phi {{{\bf{e}}}^{{\prime\prime} }}_{y}\right).$$

In this case, the path of the flipping depends on the phase difference between the spinlock pulse and the measured magnetic field (Fig. 1(c)C). The *z* component of the magnetisation after irradiation with the −*α* pulse varies, as shown in Fig. 1(c)D, due to the flip angle of the magnetisation and the phase difference of the spinlock pulse and the measured magnetic field. This leads to the change of the MR signals transmitted by the spin-echo sequence.

The behaviour of the magnetisation in the secondary magnetic resonance ${\bf{M}}=\left({{M}^{{\prime\prime} }}_{x},{{M}^{{\prime\prime} }}_{y},{{M}^{{\prime\prime} }}_{z}\right)$ is described by the following the Bloch equation:

4a $$\frac{{d{M}^{{\prime\prime} }}_{x}}{dt}=-\frac{{{M}^{{\prime\prime} }}_{x}}{{T}_{2\rho }}+\left({\omega }_{{\rm{sl}}}-\omega \right){{M}^{{\prime\prime} }}_{y}-{\omega }_{{\rm{m}}{y}^{{\prime\prime} }}{{M}^{{\prime\prime} }}_{z},$$

4b $$\frac{{d{M}^{{\prime\prime} }}_{y}}{dt}=-\left({\omega }_{{\rm{sl}}}-\omega \right){{M}^{{\prime\prime} }}_{x}-\frac{{{M}^{{\prime\prime} }}_{y}}{{T}_{2\rho }}+{\omega }_{{\rm{m}}{x}^{{\prime\prime} }}{{M}^{{\prime\prime} }}_{z},$$

4c $$\frac{{d{M}^{{\prime\prime} }}_{z}}{dt}={\omega }_{{\rm{m}}{y}^{{\prime\prime} }}{{M}^{{\prime\prime} }}_{x}-{\omega }_{{\rm{m}}{x}^{{\prime\prime} }}{{M}^{{\prime\prime} }}_{y}-\frac{{{M}^{{\prime\prime} }}_{z}-{{M}^{{\prime\prime} }}_{0}}{{T}_{1\rho }}.$$

Here, *ω*_m_ = *γB*_m_, ${\omega }_{{\rm{m}}x{\rm{{\prime} }}{\rm{{\prime} }}}={\omega }_{{\rm{m}}}\cos \phi$, and ${\omega }_{{\rm{m}}y{\rm{{\prime} }}{\rm{{\prime} }}}={\omega }_{{\rm{m}}}\sin \phi$. *ϕ* is the phase difference between the magnetic field to be measured and the spinlock pulse. *T*_1*ρ*_ and *T*_2*ρ*_ are the relaxation times. Thus, the partial spinlock sequence allows the measurement of the magnetic fields from the objects via the secondary magnetic resonance between the spinlock pulse and the measured magnetic fields.

### Simulation

We simulated the MR signal change depending on the frequency of the oscillating magnetic fields *ω* to confirm the frequency selectivity. The parameters were the magnetic field *B*_m_ = 50 nT, the spinlock frequency *ω*_sl_ = 92 Hz, and *ϕ* = 0°. The flip angle *α* was varied from 60 to 120 degrees. Then, we investigated the variation in the MR signals caused by flip angles ranging from 65 to 120 degrees in the spinlock sequence using the Bloch equation above. In this calculation, we varied *ϕ* with the fixed magnetic field *B*_m_ = 40 nT. The frequency of the magnetic field *ω*/2*π* was set to 88 Hz and the spinlock frequency *ω*_sl_/2*π* was the same. We calculated *M*_*z*_ in a single voxel when *B*_m_ was applied (${M}_{z}^{{\rm{on}}}$) and not applied (${M}_{z}^{{\rm{off}}}$) and derived the signal-change ratio ${M}_{z}^{{\rm{on}}}$/${M}_{z}^{{\rm{off}}}$. We set *T*_sl_ = 100 ms, *T*_1_ = 1300 ms, *T*_2_ = 400 ms, *T*_1*ρ*_ = 1000 ms, and *T*_2*ρ*_ = 400 ms to be tailored to experimental conditions and according to the previous report^31^. We selected the spinlock frequency *ω*_sl_ and or the frequency of the oscillating magnetic field *ω* avoiding them in a multiple of 10 Hz, because the phase of magnetisation at the end of the spinlock pulse returns to the initial phase leading to the phase-insensitive. This is an abnormal situation.

### Experiment: MRI scanner

For imaging, we employed a 7-T animal MRI scanner (BioSpin, Bruker). The fast spin-echo imaging sequence with spinlock preparation sequence was performed. The echo train length was 32, TR was 1 ms, TE was 13 ms and the scan time was 2 s per image. The imaging direction was axial. The field of view (FOV) was 4.0 cm × 4.0 cm, the matrix was 64 × 64, and the thickness of each slice was 2 mm. A function generator (AFG3102C, Tektronix) was synchronised with the MRI scanner to apply sinusoidal voltages to the phantom via a 20-kΩ resistor during the spinlock pulses. The imaging slice was a plane including the loop coil and the current-dipole electrodes. *T*_sl_ was fixed to 100 ms. The spinlock frequency *ω*_sl_ was set to around 90 Hz, but the actual value was determined by the averaged RF amplitude over FOV. We measured MR signals when *B*_m_ was applied *S*^on^ and was not applied *S*^off^ and calculated the signal-change ratio *S*^on^/*S*^off^.

### Experiment: loop-coil phantom

**Figure 2:**
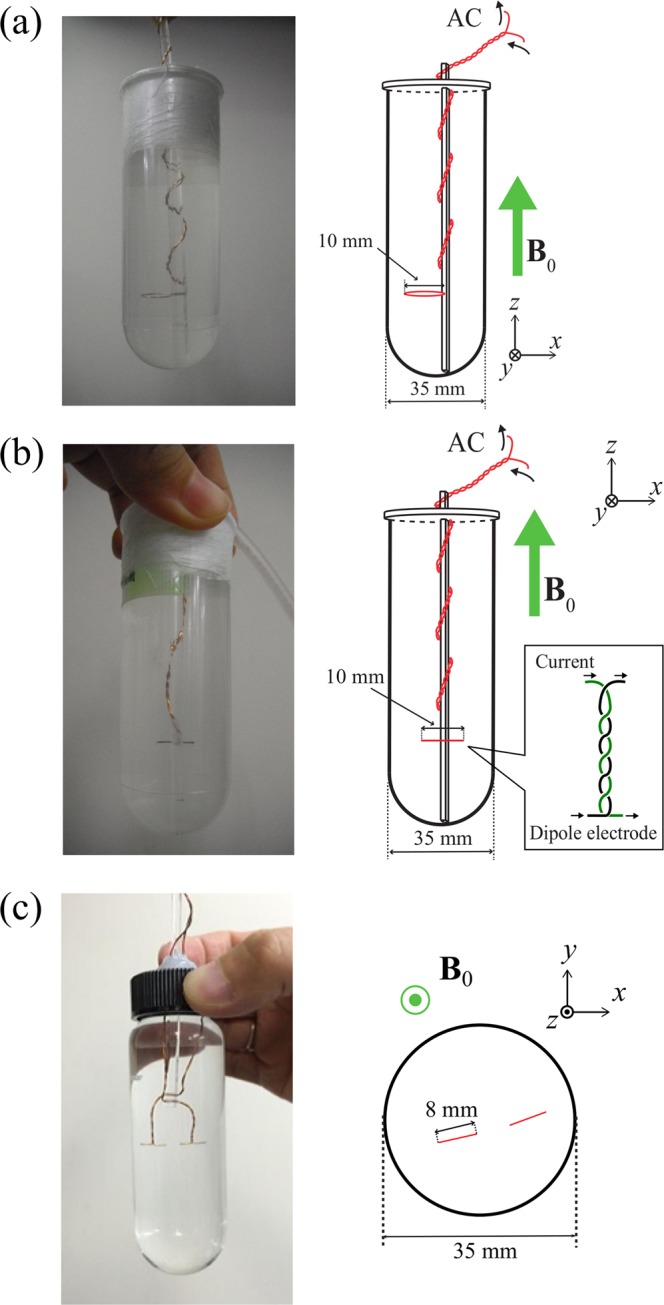
Phantoms used in experiments. (**a**) Photograph (left) and illustration (right) of the loop-coil phantom (**b**) Photograph (left) and illustration (right) of current-dipole phantom (**c**) Photograph (left) and illustration (right) of current-dipole phantom containing two dipole electrodes. The coil and the dipole electrodes were held with an acrylic rod in a normal saline solution.

In our first round of tests, we used a loop-coil phantom for the simplicity of calculating the magnetic field around a loop current. Figure 2(a) shows a photograph of the loop-coil phantom that we used in this experiment. The loop coil was made of 0.25-mm copper wire coated with polyurethane with a diameter of 10 mm. To prevent the generation of a stray magnetic field, all copper wire other than the loop part was twisted, and the loop was fixed to an acrylic rod. The loop coil was placed in a plastic tube with a diameter of 35 mm. The plastic tube was filled with saline-added contrast medium (Magnevist, Bayer) until the *T*_1_ relaxation time was about 1300 ms and was then sealed with a lid and paraffin film. The phantom was placed in the bore of the MRI scanner with the plane of the loop coil perpendicular to *B*_0_. The region of interest was 2 × 2 voxels at the centre of the loop coil.

First, we checked the frequency selectivity of the partial spinlock sequence, varying the frequency *ω*/2*π* of *B*_m_, while *ω*_sl_/2*π* was fixed to 92 Hz. The flip angle *α* was 100°, the initial phase *ϕ* was 0°, and *B*_m_ was 47 nT in the centre of the loop coil, which was calculated by the Biot-Savart law. Next, we set the spinlock frequency as 88 Hz. The initial phase *ϕ* of the measured magnetic field *B*_m_ was varied from 0 to 360°. The frequency of *B*_m_ was 88 Hz and the amplitude of *B*_m_ at the centre of the loop coil was estimated as 40 nT by the Biot-Savart law.

### Experiment: Current-dipole phantom

A current-dipole phantom was used to simulate the neuromagnetic fields generated by the distributed electric currents inside the human head. Figure 2(b) shows the current-dipole phantom used in the experiment. The electrode was made of two 0.25-mm copper wires coated in polyurethane. The wires were wound and angled to form a current-dipole electrode of 1 cm long. The ends of the current-dipole electrode were polished with sandpaper to remove the polyurethane. Like the loop-coil phantom, all wires except for the dipole electrode were twisted to suppress the generation of magnetic fields. The electrode was immobilised with an acrylic rod and placed in a saline solution to set the *T*_1_ relaxation time to about 1300 ms with a contrast medium. The diameter of the plastic tube was 35 mm and it was sealed with a lid and paraffin films. The phantom was placed in the MRI scanner so that the dipole was perpendicular to **B**_0_.

We examined the *ϕ* dependence on the MR signal change. *B*_sl_ was set to make the spinlock frequency *ω*_sl_/2*π* = 95 Hz. The phase difference *ϕ* was varied from 0 to 360°, and *α* was fixed to 110°. The input voltage of the dipole electrode was *V* = 20 V_pp_ with a frequency of 95 Hz, and the current-dipole moment was about 3 *μ*Am. The regions of interest were 4 voxels 2 mm away from the dipole electrode. We also examined a tiny magnetic field detection. *ω*_sl_/2*π* and *ω*/2*π* were set to 96 Hz, and *α* and *ϕ* were set to 110° and 0°, respectively. The current-dipole moment we evaluated was 500 nAm, 250 nAm, 100 nAm and 50 nAm. As a control, we acquired the MR images without the oscillating magnetic fields. In each case, we acquired 10 images.

To simulate a situation multiple signal sources, we fabricated a phantom containing two dipole electrodes, 8 mm in length and with a diameter of 0.25 mm, shown in Fig. 2(c). The diameter of the phantom was 35 mm. Those dipole electrodes were connected in series with 20-kΩ resistors, and sinusoidal voltages were applied with *V* = 10 V_pp_, independently. Each current dipole was about 1.4 *μ*Am. The initial phases of the dipoles *ϕ*_1_ and *ϕ*_2_ were set to 0° or 180°, *ω*_sl_/2*π* and *ω*/2*π* were set to 95 Hz, and *α* was set to 100°.

## Results

### Simulation

**Figure 3:**
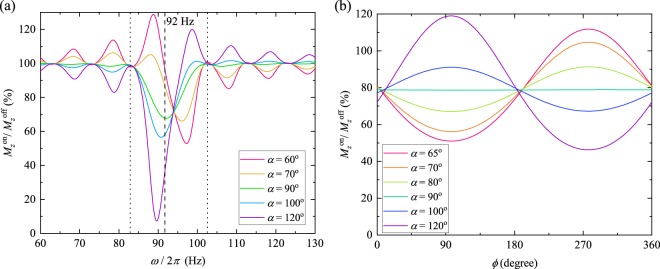
Simulated MR signal changes as a function of (**a**) the frequency of the measured magnetic fields and (**b**) the initial phase *ϕ* with various *α*. (**a**) The flip angle *α* was varied from 60 to 120 degrees. The magnetic field *B*_m_ = 50 nT, the spinlock frequency *ω*_sl_/2*π* = 92 Hz, and *ϕ* = 0°. (**b**) The magnetic field *B*_m_ = 40 nT, and the frequency *ω*/2*π* and the spinlock frequency *ω*_sl_/2*π* were 88 Hz.

 Figure 3(a) shows the simulated MR signal changes as a function of the frequency *ω*/2*π* of the measured magnetic fields with *α* ranging from 60° to 120°. In the case of *α* = 90°, the minimum signal change was 67.6% at 92 Hz, and the full width at half maximum (FWHM) was 9 Hz. By changing the *α*, the minimum value, the minimum frequency and the FWHM changed. At *α* = 100°, the minimum value was 56.5% and the minimum frequency was 90.8 Hz with the FWHM at 7.8 Hz. At *α* = 120°, the minimum value was 7.4% and the minimum frequency was 89.6 Hz with the FWHM at 5.4 Hz. The shift of the minimum frequency was caused by the longitudinal component of the magnetisation, which causes a phase difference in the rotating frame, which is a frequency shift in the laboratory frame. The shift, however, occurred between two nodes, 82.5 Hz and 102.5 Hz, which was the same in the case of *α* = 90°.

The change in the MR signal as a function of *ϕ* was simulated with the flip angle *α* ranging from 65–120°, as shown in Fig. 3(b). The signal change varied sinusoidally at all values of *α*. When increasing *α* from 65 to 120°, the signal-change curves were inverted in their local maximum and local minimum through *α* = 90°. By selecting *α*, we can enhance the signal-change ratio compared to *α* = 90°.

### Experiment: loop-coil phantom

**Figure 4:**
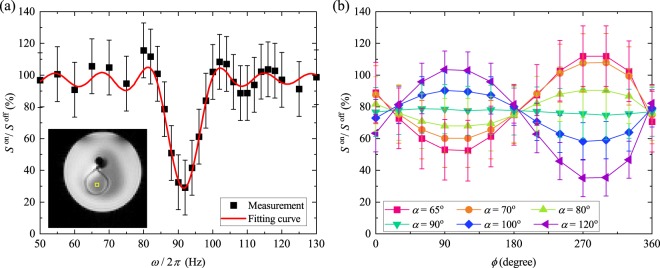
Measured MR signal change as a function of (**a**) the frequency of the measured magnetic fields and the fitting curve with the fitting parameters of *α*, *ω*_sl_ and *B*_m_ and (**b**) the initial phase *ϕ* with different *α* ranging 65° to 120°. (**a**) The set values were *B*_m_ = 47 nT, *ω*_sl_/2*π* = 92 Hz, and *α* = 100°. The estimated values from the fitting were *B*_m_ = 37 nT, *ω*_sl_/2*π* = 92 Hz, and *α* = 98°. (**b**) The magnetic field *B*_m_ = 40 nT, and the frequency *ω*/2*π* and the spinlock frequency *ω*_sl_/2*π* were 88 Hz. The error bars show the standard deviation of the MR signal in the phantom without the oscillating magnetic fields (inset). The yellow box in the inset shows the region of interest.

The frequency selectivity was demonstrated in Fig. 4(a). To reduce the effect of the frequency shift, we selected *α* = 100° in this experiment. The error bars indicated the standard deviation of the MR signal in the phantom without oscillating magnetic fields, as shown in the inset. The experimental data were fitted with the Bloch equation using *α*, *ω*_sl_ and *B*_m_ as the fitting parameters. According to the fitting curve, the parameters were estimated as *α* = 98° and *ω*_sl_/2*π* = 92 Hz. These values agreed well with the set values. However, *B*_m_ was estimated as 37 nT. *B*_m_ slightly differed from the calculated value. The error was about 21%. This could be due to the slight lean of the loop coil and the difference of *T*_1*ρ*_ and *T*_2*ρ*_. The FWHM from the measurement was 9.7 Hz, which was broader than the calculation. The relaxations, therefore, could affect the phase dispersion of the magnetisation.

 Figure 4(b) shows the measured MR signal change with *ω*_sl_/2*π* = 88 Hz as a function of *ϕ*. According to the Biot-Savart law, the calculated *B*_m_ was 40 nT at the centre of the loop coil in the experiment. We varied *α* in this experiment. The inset shows an MR image of the loop-coil phantom without *B*_m_ indicating the region of interest. The error bars show the standard deviation of the MR signal in the phantom without the oscillating magnetic fields. It, therefore, indicates the inhomogeneity of the RF fields. The results coincided with the simulation in Fig. 3(b). In the case of *α* = 120°, the data seemed to shift downward. The difference was caused by the fluctuation of RF fields. In high *α*, a slight change of the spinlock frequency leads to a drastic change of the signal-change ratio. When measuring the spatial homogeneity of the RF fields by the B1 map, the strength of the fields at the centre of the loop coil was 50% larger than the set value displayed at the console. In the experiments, we corrected the value, but a small fluctuation could still occur.

### Experiment: current-dipole phantom

**Figure 5:**
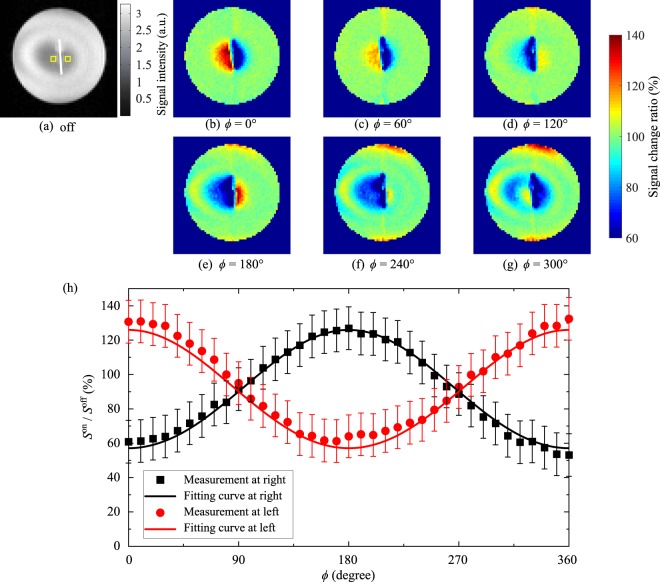
MR images of the dipole phantom with different initial phases. (**a**) *B*_m_ = 0 nT, (**b**) *ϕ* = 0°, (**c**) *ϕ* = 60°, (**d**) *ϕ* = 120°, (**e**) *ϕ* = 180°, (**f**) *ϕ* = 240°, and (**g**) *ϕ* = 300°. (**h**) MR signal change as a function of the initial phase *ϕ* to the right side and the left side of the current-dipole electrode. The error bars show the standard deviation of the MR signal in the phantom without the oscillating magnetic fields. The electrode was drawn with a white line and the regions of interest were shown with yellow boxes in (**a**). The fitted values were *ω*_sl_/2*π* = 95 Hz, *α* = 112°, and *B*_m_ = 50 nT in (**b**–**g**).

 Figure 5 shows MR images of the current-dipole phantom with *ϕ* varied from 0 to 300° and the MR signal changes as a function of *ϕ*. In Fig. 5(a), we show the regions of interest with yellow boxes. There was a region of low signal intensity around the dipole electrode despite no oscillating magnetic fields. This was due to the inhomogeneity of the RF fields. By increasing *ϕ* from 0 to 300°, the positions at which the MR signal increased and decreased inverted, as shown in Fig. 5(b–g). In the cases of *ϕ* = 0° and 60°, the MR signals decreased to the right side of the dipole electrode, while the signals decreased to the left side in the cases of *ϕ* = 180° and 240°. For the analysis, we averaged the signal intensity in 2 × 2 voxels around each side. Figure 5(h) shows the MR signal-change as a function of *ϕ*. The error bars show the standard deviation of the MR signal in the phantom without the oscillating magnetic fields. The data were fitted to theoretical curves with fitting parameters of *α* and *B*_m_. The fitted values were *α* = 102° and *B*_m_ = 50 nT. The dependence of the signal changes in the right and left regions of the dipole electrode on the initial phase *ϕ* were inverted due to the 180° phase reversal in the left and right regions of the dipole electrode. Figures 3(b) and 4(b) also support this finding.

**Figure 6:**
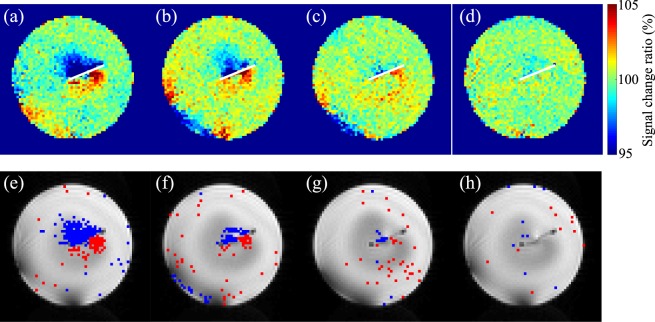
MR images of tiny magnetic field measurements. *ω*/2*π* and *ω*_sl_/2*π* were 96 Hz, and *α* and *ϕ* were set to 110° and 0°, respectively. Each image was averaged with 10 images. The current-dipole moment was (**a**) 500 nAm, (**b**) 250 nAm, (**c**) 100 nAm, and (**d**) 50 nAm. The white line indicated the dipole electrode. Two-sided *t*-test results of (**a–d**) were shown in (**e–h**), The threshold was *p* < 0.01. The negative and positive signal changes were indicated with blue and red pixels, respectively.

Figure 6 shows 10-times averaged MR images of tiny magnetic field measurements. Figure 6(a–d) were signal-change ratios with current-dipole moments of 500 nAm, 250 nAm, 100 nAm and 50 nAm, respectively. Figure 6(e–h) were the results of *t*-test (two-sided, *p* < 0.01) of Fig. 6(a–d). The blue and red pixels were negative and positive signal changes, respectively. In the cases of 500 nAm, 250 nAm and 100 nAm, there were positive and negative signal changes in both sides of the dipole electrode, although there was no signal change in the case of 50 nAm. Calculating the magnetic fields in the voxel with a significant difference in Fig. 6(g) by the Biot-Savart law, the magnetic field detected by the method was estimated to about 0.8 nT.

**Figure 7:**
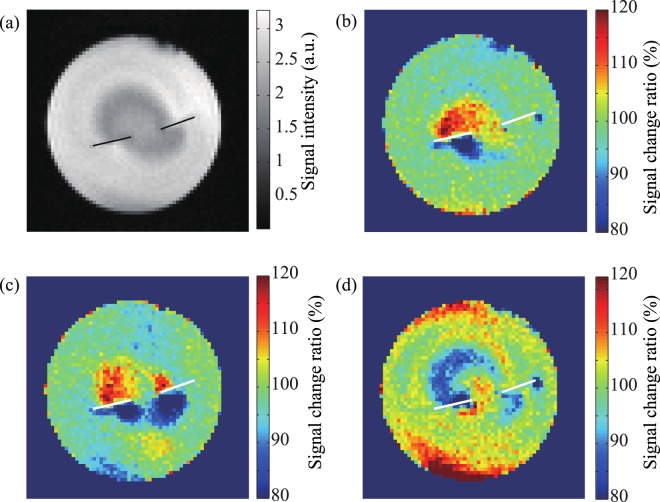
MR images of the phantom containing two dipole electrodes. The current dipole moments were 1.4 *μ*Am, and the frequency *ω*/2*π* was 95 Hz. *ω*_sl_/2*π* and *α* were 95 Hz and 100°, respectively. (**a**) Dipole electrode 1 (left) and 2 (right) turning off, (**b**) Dipole electrode 1 turning on with *ϕ*_1_ = 0°, and electrode 2 turning off, (**c**) Dipole electrode 1 turning on with *ϕ*_1_ = 0° and electrode 2 turning on with *ϕ*_2_ = 0°, and (**d**) Dipole electrode 1 turning on with *ϕ*_1_ = 180° and electrode 2 turning on with *ϕ*_2_ = 0°.

 Figure 7 shows MR images of the phantom containing two dipole electrodes. In this case, we changed the initial phases of both dipole electrodes, *ϕ*_1_ and *ϕ*_2_. Figure 7(a) was taken as a reference with the electrodes turning off. Figure 7(b) shows the MR image with electrode 1 turning on with *ϕ*_1_ = 0° and electrode 2 turning off, and Fig. 7(c) shows the MR image taken with both electrodes turning on with *ϕ*_1_ and *ϕ*_2_ = 0°. The MR signal changed only around electrode 1 in Fig. 7(b). The MR signals changed around both electrodes in Fig. 7(c). In Fig. 7(d), the applied voltages of electrodes 1 and 2 had a 180° phase difference, so the MR signals decreased in the upper region around electrode 1 and the lower region of electrode 2. The spatial inhomogeneity of *B*_sl_ and the distributed currents in the saline, however, made the analysis challenging. As similar to in Fig. 7(a), there was a darker region at the centre of the phantom caused by the inhomogeneity in Fig. 7(d). Furthermore, two current-dipole moments with opposite phases facing each other caused a complicated distribution of the currents in the phantom. There, therefore, was a large signal change at the edges of the phantom. To acquire coherent images of neuronal activity, we should suppress such inhomogeneity and analyse the distributed currents in the human brain.

## Discussion

We found that the MR signals varied periodically depending on the initial phase of the measured magnetic fields. The signal variations in the spinlock sequence depended only on *B*_m_ when *α* = 90°, while they depended on both *B*_m_ and *ϕ* when *α* ≠ 90°. This method detects the magnetic fields in the *z*-direction, because the magnetic fields with low frequency in the *x*-*y* plane do not affect the MR signals. Furthermore, the spinlock sequence intrinsically has frequency selectivity. The combination of the signal changes with *α* = 90° and *α* ≠ 90°, therefore, could allow the detection of functional connectivity^29^. The partial spinlock sequence with *α* ≠ 90° is sensitive to both the amplitude and the phase of the measured magnetic fields. To determine the amplitude of the magnetic fields, it is effective to apply the SIRS with *α* = 90°, which is insensitive to the phase. Furthermore, this phenomenon indicates the feasibility of detecting small magnetic fields with the appropriate selection of *α* and *ϕ*. According to Figs. 3(b) and 4(b), the signal change was enhanced with *ϕ* = 90° and 270°. The value, however, was dependent on the spinlock frequency and the frequency of the oscillating magnetic fields. In the case of *α*, the larger and smaller the flip angle was, the greater the signal change was. This means that the magnetisation component, which is not spin-locked, is critical in the method. As shown in Fig. 3(a), a large *α*, however, causes a large fluctuation of the signal change. Furthermore, the side lobe of the spinlock spectra increased with an increase in *α*. This will be the false detection. This result suggests that the non-right flip angles will enhance the signal changes of the spinlock sequence when *ϕ* is known, such as steady-state evoked fields. Most of the measurements in this study, however, uses strong oscillating magnetic fields in the order of tens of nT. Our previous study shows that the SIRS could detect 200-pT magnetic fields with a *t*-test and the smallest magnetic field with the partial spinlock sequence was 0.8 nT in this study. The neuromagnetic fields are estimated in the range of 0.1–1 nT^32^, so the method itself can detect neuromagnetic fields. Due to the SNR limitation of our MRI scanner, it, however, was challenging to obtain clear trends with weak magnetic fields. We, therefore, must improve the SNR of the scanner.

In the current-dipole phantom measurements, we measured both coherent signals and phase-reversed signals. The signal change varies drastically at the position of the current dipoles. We found that the positions of the current dipoles could be estimated to the positions that the sign of the signal change was reversed because the method directly measured magnetic fields, unlike the BOLD-fMRI. The measurements with two dipole electrodes applying opposite phases, however, showed a complicated distribution of the signal change, especially at the edges of the phantom. In the experiment, the diameter of the phantom was 35 mm due to the limitation of the bore size of the scanner. This makes the distributed currents complex. We might require solving the sophisticated inverse problem. The size of the human brain, however, is ten times larger than that of the phantom. It, therefore, is thought that the distributed currents do not affect the signal change in the human brain measurements. Our method is based on the SIRS; therefore, the spatiotemporal resolution is the same as the SIRS. The spatial resolution of the SIRS is similar to the BOLD-fMRI because it is limited by the voxel size. The activation area, however, is accurate compared to the BOLD effects. On the other hand, the temporal resolution is limited by the duration of the spinlock pulse and the acquisition sequence. The spinlock pulse is usually several tens to hundreds ms. In the SIRS, we can apply most of the fast acquisition sequences. Furthermore, the neural magnetic fields occur immediately with brain activities, and the method would estimate the area of neural activities more accurately with the current-dipole model.

Witzel *et al*. reported that the detectable current-dipole moment was as small as 56 nAm by SIRS with a 3-T MRI scanner^16^, and Halpern-Manners *et al*. suggested the SIRS could detect 0.46-nT magnetic fields with a 7-T scanner^17^. These values are slightly better than those of ours. The voxel size of the former study, however, was about 50 times larger than in our study. That of the latter, however, was about 20 times smaller than ours. In this case, they used a spin-echo imaging sequence, so the measurement time was about 1 minute. We, therefore, consider that our results were consistent with their study. SLOE also have been studied energetically. Sheng *et al*. reported that the smallest magnetic field detected by SLOE was lower than 0.1 nT in a phantom and 0.5 nT in a rat brain with a 3-T scanner^33^. In this case, they wound a coil around the heads of rats to generate oscillating fields. They compared SLOE with SIRS, and found that SLOE had a higher contrast than SIRS. The results were better than ours were, although the voxel size was about 5 times larger than our results. Chai *et al*. studied SLOE with a 7-T scanner comparing SIRS and BOLD-fMRI of rat brains with optical stimulation^28^. They also reported that the sensitivity of SLOE, 0.5 nT, was 10 times better than SIRS and that SLOE could detect the phase (0° or 180°) of the magnetic fields. The voxel size was 3.5 times smaller than ours. The BOLD-fMRI, however, could observe the activation area in the rat brains, but both SLOE and SIRS could not. They concluded that sensitivity should be further improvement. The other method suggested by Truong *et al*. was examined under a 3-T scanner^19^. The sensitivity was reported at 0.06 nT in a phantom measurement, although the voxel size was 40 times larger than in our study. They also evaluated human brains; however, the activation area was different from the BOLD-fMRI. Further study, therefore, is necessary to understand the phenomenon. Considering all the factors involved, SLOE and Truong’s method are promising candidates for the neural magnetic field-dependent fMRI. They, however, are sensitive to the amplitude and the phase of the magnetic fields. To analyse the MR signal quantitatively, they, therefore, require a phase-insensitive sequence. In our method, it is easy to use the phase-insensitive sequence by changing the flip angle *α*.

For biomagnetic field imaging, the B0 and B1 inhomogeneity in the spinlock sequence could be an issue. There were darker regions in the centre of the phantoms in the experiments. In the experiments, the proper RF amplitudes were calculated from the average of the MR signals over FOV. The actual RF amplitude at the region of interest, therefore, differed slightly from the set value. That is why the spinlock frequencies and flip angles were different in each measurement. To avoid the effects, an interleaved 180° pulse or alternating the phase of the spinlock pulse at the halfway point would be promising. These methods have a good track record on the spinlock sequences^18,34^, and do not affect phase-detection because the magnetisation component that is not spin-locked turns just from $+z{\prime}$ to $-z{\prime}$. The other way to suppress the inhomogeneity is to introduce adiabatic pulses^35^. This method provided homogeneous *T*_1*ρ*_ maps with reduced artefacts. Furthermore, Zu *et al*. reported that the low spinlock frequency around 100 Hz causes *R*_1*ρ*_ = 1/*T*_1*ρ*_ dispersion in solute and water^36^. According to our previous study, *R*_1*ρ*_ is an important parameter to determine the signal-change ratio^30^. We, therefore, should assess the effects of the dispersion in the case with a contrast agent, although our method could be used without any contrast agents. Furthermore, the BOLD effects could generate artefacts with this method, although the BOLD effect appears with some delay after neural activity^16^. The BOLD effects occur in the acquisition sequence despite the presence or absence of the spinlock preparation sequence, that is, we might not distinguish the MR signal suppression caused by the SIRS from that caused by the BOLD effects. In future studies, we, therefore, must evaluate the method with animal and human subjects. Another approach is using the low and ultra-low-field MRIs^37–39^, which are affected by neither the BOLD effect nor magnetic susceptibility. The signal changes by the (partial) spinlock sequence do not depend on the strength of the static fields. The signal to noise ratio,therefore, is attributed to the intrinsic noise of the MRI scanners. This leads to the suggestion that the method could be one of the candidates of the low-field fMRI.

## Conclusion

We investigated the plausibility of a spinlock module with non-right RF pulse for functional connectivity measurements both in Bloch simulations and a phantom study. By the simulation, we showed the frequency selectivity of the method and found that non-right flip angles in the spinlock sequence can increase the variation about 2–3 times in the MR signal and depend on the initial phase of the measured magnetic fields. The MR signal changes periodically as a function of the initial phase. We confirmed the simulation results in the experiments with a loop-coil phantom. In addition, dipole phantom experiments indicated that the signals from two sources could be detected and their phase difference distinguished. These results demonstrate the possibility of the fMRI measurements sensitive to the phase of oscillating magnetic fields using the partial spinlock sequence.

## Data Availability

The data are available from the corresponding author on reasonable request.
